# *Aspergillus fumigatus*—What Makes the Species a Ubiquitous Human Fungal Pathogen?

**DOI:** 10.1371/journal.ppat.1003743

**Published:** 2013-12-05

**Authors:** Kyung J. Kwon-Chung, Janyce A. Sugui

**Affiliations:** Molecular Microbiology Section, Laboratory of Clinical Infectious Diseases, National Institute of Allergy and Infectious Diseases, National Institutes of Health, Bethesda, Maryland, United States of America; Duke University Medical Center, United States of America

## Introduction

*Aspergillus fumigatus*, the major cause of life threatening invasive aspergillosis (IA), is a ubiquitous saprophytic fungus to which humans are exposed daily in most parts of the world. The infection is initiated by inhalation of conidia, which are cleared quickly in a normal host but can cause invasive disease in immunocompromised individuals [1], [2]. The following features make *A. fumigatus* a ubiquitous pathogen: 1) survival and growth in a wide range of environmental conditions, 2) effective dispersal in the air, 3) physical characteristics that allow conidia to reach the distal airways, and 4) swift adaptability to the host environment. The biology, pathogenesis, molecular biology, and virulence factors of *A. fumigatus* have been exhaustively reviewed [2]–[8]. This brief article focuses on how *A. fumigatus* is equipped with the features necessary for a ubiquitous pathogen.

## *Aspergillus fumigatus* Is Equipped to Survive and Propagate Successfully under a Wide Range of Environmental Conditions

**Figure 1:**
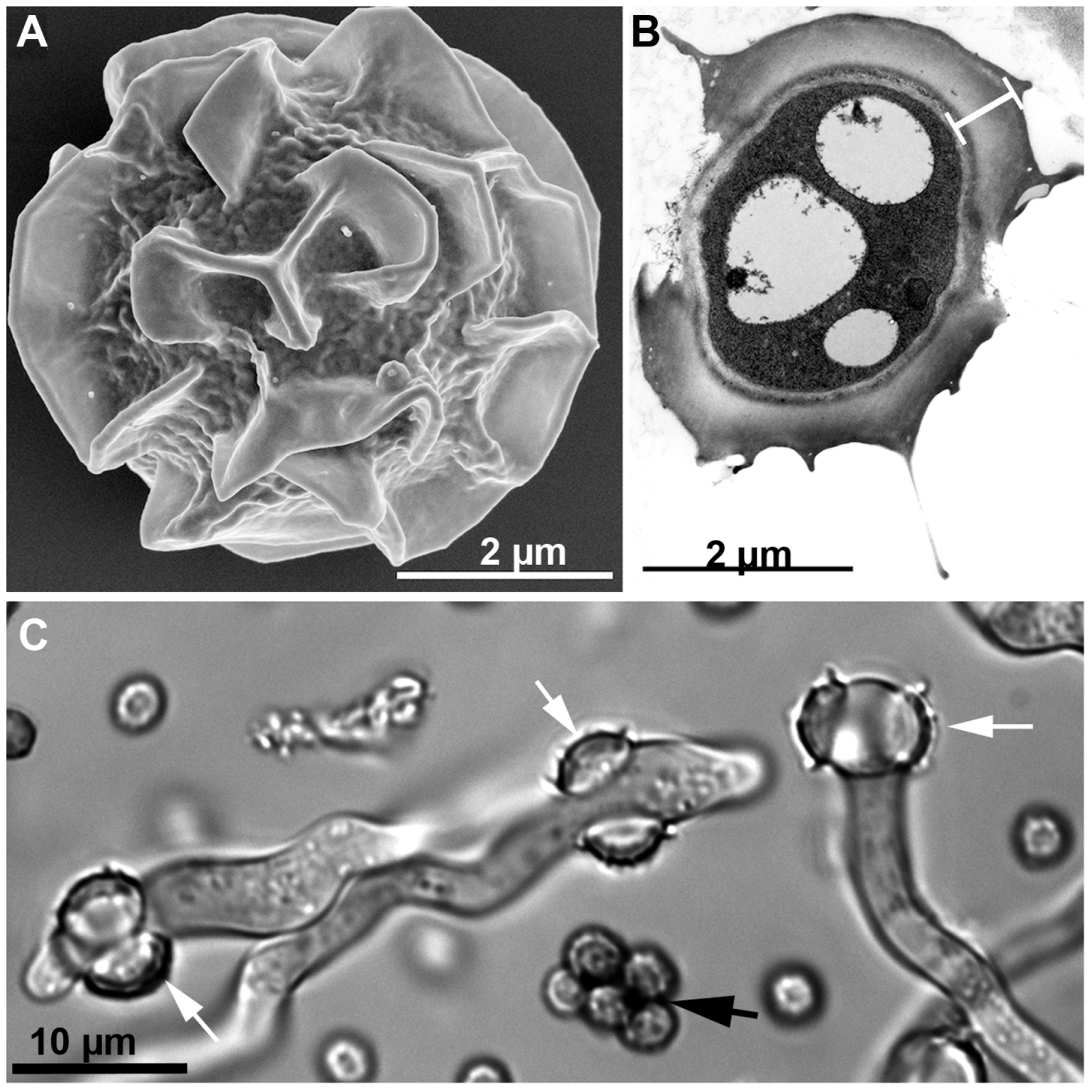
*Aspergillus fumigatus* ascospores. A) SEM image of an ascospore produced by mating between two compatible strains. Courtesy of Bryan Hansen. B) TEM image of an ascospore cross-section showing an unusually thick wall (white bar) composed of an electron-dense inner wall covered by a thick outer wall. Courtesy of Mones Abu-Asab. C) DIC image of germinating ascospores (white arrows) and dead conidia (black arrow) after 30 min incubation at 70°C.

In most parts of the world, *Aspergillus fumigatus* can be isolated from a wide variety of substrates throughout the year. Although *A. fumigatus* grows optimally at 37°C and a pH 3.7 to 7.6, it can be isolated wherever decaying vegetation and soil reach temperatures range between 12° and 65°C [9] and the pH ranges between 2.1–8.8 [10]. *A. fumigatus* was found to be the dominant fungus in garden and greenhouse soil that comprised 35 to 70 percent of the total numbers of colony-forming fungi [10]. As an efficient recycler in nature, *A. fumigatus* possesses a versatile metabolism that meets its nutritional requirements under different environmental conditions [11]. The presence of numerous glycosylhydrolases [6] and a group of extracellular proteinases in the *A. fumigatus* genome attest to the ability of the fungus to grow by degradation of polysaccharides from plant cell walls and acquire nitrogen sources made available by degradation of proteinacious substrates [8]. Self-heating compost heaps are major environmental sources of *A. fumigatus* due to its pronounced thermotolerance. One study found 100,000 colony-forming units (cfu)/gram/dry weight of compost [12], and compost piles of chipped leaves and branches may yield massive and almost pure cultures of *A. fumigatus* [1]. The thermotolerance of *A. fumigatus* is even more remarkable in the ascospores, the propagules produced in the sexual cycle. The ascospores of *A. fumigatus* (Figure 1A) are protected by an extraordinarily thick wall (Figure 1B) compared to those of other aspergilli such as *A. nidulans* [13]. The ascospores of *A. fumigatus* germinate after heating at 70°C for 30 min [14] (Figure 1C) and should survive at core temperatures of the compost pile that can reach ≥70°C [2].

Although *A. fumigatus* fails to grow at temperatures below 12°C, its conidia can tolerate stresses imposed by freezing for prolonged periods. Depending on the strain, conidia can survive in liquid nitrogen for up to 18 years [9]. Although a few genes associated with fungal growth at ≥48°C have been characterized, the genetic systems involved in survival and growth under extreme temperatures remain unidentified [15]. *A. fumigatus* conidia can also tolerate dehydration for prolonged periods, surviving for more than 60 years when lyophilized, and the conidia that had been maintained in anhydrous silica gel survived for more than 25 years (unpublished data).

The wide distribution of *A. fumigatus* in nature may also be due to the presence of successful defense systems such as the production of potent secondary metabolites and efflux pumps. The *A. fumigatus* genome contains 22 secondary metabolism gene clusters [11] and 16 different secondary metabolites have been identified [16], including gliotoxin, a broad range antimicrobial [17]. *A. fumigatus* possesses a higher number of ABC transporters than its close genetic relative, *Aspergillus fischerianus* [15]. The *A. fumigatus* genome is also rich in specific enzymes such as catalases, superoxide dismutases, and glutathione transferases for the detoxification of reactive oxygen species (ROS) [8], [18]. All these features equip *A. fumigatus* to survive and propagate in conditions that are detrimental to a broad range of other environmental organisms.

## *Aspergillus fumigatus* Conidia Are Dispersed More Efficiently in the Air Than Those of Most Other Molds

**Figure 2:**
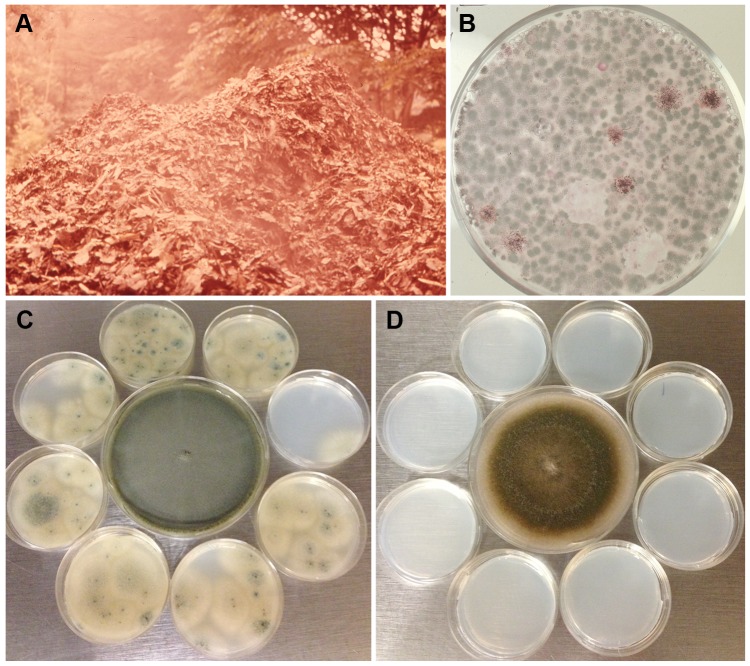
Dispersibility of *A. fumigatus* conidia. A) A cloud of aerosol released in the air after turning of a compost pile located in Maryland, USA. B) A malt extract agar plate exposed to the air for a minute at the site and incubated for a few days at 37°C grew predominantly *A. fumigatus* colonies (both pictures were taken by the late Dr. Chester Emmons). C) Eight small sterile agar plates of *Aspergillus* minimal medium were placed around a seven-day-old culture of *A. fumigatus* strain B-5233 (center) in a class 2 biosafety cabinet. In the absence of air flow the lids of all the plates were removed for 24 h. The small plates were then incubated for three days at 37°C. D) The same procedure as in C except that the small plates were exposed to the culture of a ten-day-old *A. nidulans* strain RYC13B (center). *A. fumigatus* conidia dispersed to the surrounding small agar plates while none was evident for the *A. nidulans* strain.

*Aspergillus fumigatus* conidia accumulate 1,8-dihydroxynaphthalene melanin in their cell wall, have a blue-green color [19], [20], and are notorious for their high dispersibility. The slightest air current can cause conidia to disperse due to their remarkable hydrophobicity, and these airborne conidia are protected from ultraviolet irradiation due to the melanin in their cell wall [20]. One study has estimated the emission rate of *A. fumigatus* conidia from an undisturbed compost pile to be 8–11×10^3^ cfu/m^2^/s at the mean wind speed of 1 m/s [21], which indicates how efficiently conidia are dispersed with the slightest agitation. Figure 2A shows an aerosol cloud over a disturbed compost pile. A majority of the microbial growth on a plate of agar medium briefly exposed to the air at the site was that of *A. fumigatus* (Figure 2B). Although all fungal spores produced on aerial hyphae or conidiophores are hydrophobic, the degree varies from mild to highly hydrophobic [22] which impacts the efficiency of spore dispersibility. *A. fumigatus* conidia are considerably more hydrophobic than those of other aspergilli such as *A. nidulans*. This requires more caution in the handling of *A. fumigatus* cultures than other fungi to prevent contamination of surrounding areas in the laboratory (Figure 2C, D). Conidial hydrophobicity is conferred by the surface rodlet layer encoded by the *rodA* gene [23]. In addition to dispersal of airborne conidia, conidia imbedded in soil may also be effectively transported from one place to another by swarming soil bacteria such as *Paenibacillus vortex*. *P. vortex* facilitates the dispersal of *A. fumigatus* more efficiently than other fungal species that have similarly sized conidia such as *Penicillium expansum* or *P. citrinum* [24]. Conidial surface proteins are crucial for the passive dispersal of *A. fumigatus* by the bacteria since proteinase K treatment of conidia abolished the conidia-bacterial interaction. Undoubtedly, *A. fumigatus* conidia are also being passively dispersed via rodents, insects, and worms but the impact of *A. fumigatus* spread by these means has not been studied.

## Physical Characteristics of Conidia That Contribute to Respiratory Tract Disease

Fungal spores account for a significant proportion of the aerosol particle mass that the human respiratory system is exposed to daily. Airborne fungal spores exist in various sizes and any spore with a size of ≥5.0 µm (diameter) is too large to reach the lower airways [25] where systemic infection is primarily initiated. *A. fumigatus* conidia are globose to subglobose with a size (2.0–3.0 µm in diameter with extremes up to 3.5 µm) adequate to bypass mucociliary clearance and reach the lower airways. The average adult inhales more than 100 *A. fumigatus* conidia daily since the conidial concentration in the air indoors or outdoors is estimated to be 1–100 conidia/m^3^ [4]. Conidial size does not change significantly with increased relative humidity from 30% to 90% [26], and so airborne conidia maintain an optimum size for reaching the lower airways regardless of the relative humidity. Melanin in the conidial wall offers protection from ROS while also enabling resistance to lysis by host cells [4]. *A. fumigatus* conidial surface contains more exposed negatively charged sialic acid residues than other *Aspergillus* species and sialic acid partly mediates binding to basal lamina proteins of the host [27]. *A. fumigatus* conidia, therefore, may adhere to the epithelium of airways and alveoli more effectively than other fungal species with similarly sized airborne spores.

## *Aspergillus fumigatus* Conidia Germinate and Adapt Readily to the Immunocompromised Host Environment

*Aspergillus fumigatus* conidia that reach the alveoli are unable to withstand the immune assault mounted by normal hosts because the fungus lacks specialized virulence factors [6]. However, patients who are undergoing organ transplantation, cancer chemotherapy, or have chronic granulomatous disease (CGD) as an underlying condition are highly susceptible to infection by the fungus because the inhaled conidia can efficiently adapt their physiology to the altered host environment. A review of 146 autopsies at the National Institutes of Health over a 22-year period showed no firm link between hospital exposure and an increased incidence of invasive aspergillosis. There was, however, a clear link between cancer chemotherapy regimens and increased incidence [28]. This indicates that adaptability of *A. fumigatus* to the human environment, though successful, is secondary to the host immune status. Inhaled conidia readily germinate at the mammalian body temperature since 37°C is the optimum temperature for both germination and growth. Conidia shed the hydrophobin layer and swell in 4 h to germinate into short hyphae by 6–8 h at 37°C in vitro as well as in immunocompromised mammalian tissue [29]. During this early growth period, *A. fumigatus* responds to the stress imposed by the host environment by utilizing a highly coordinated gene expression program that enables adaptation to iron limitation, nitrogen and glucose deprivation, alkaline stress, and other unfavorable conditions [29]. One of the features during early infection in mice is the activation of gliotoxin biosynthesis [29]. Since gliotoxin is immunosuppressive and cytocidal [17], it can be speculated that the fungus benefits from nutrients released by the gliotoxin-destroyed host cells. Presence of the toxin in sera of patients infected with *A. fumigatus* suggests its involvement in the adaptation to the host environment [17]. How efficiently *A. fumigatus* cells can sense and respond to the host environment has been shown by clear differences in transcriptional profiles between conidia exposed to the neutrophils of normal host compared to those from patients with CGD, which are defective in ROS production [30]. All these features indicate that, being equipped to grow in a wide range of unfavorable conditions in nature, *A. fumigatus* finds the immunocompromised host environment just another adverse condition to which it can successfully adapt.

## Concluding Remarks

Among the over 200 species of *Aspergillus*, *A. fumigatus* is the best at meeting the four features discussed in this review. Since innate immunity protects against *Aspergillus*, the reason for the wide spread of IA caused by *A. fumigatus* is due to the global distribution of both the fungus and an increase in susceptible hosts. However, only a portion of the high-risk population, such as those with stem cell transplantation or CGD, develop IA despite daily exposure to the fungus. This suggests that a genetic risk associated with aspergillosis may exist in IA patients in addition to their underlying immunosuppressive condition. Although several studies on the role of immune-related gene SNPs of both donors and recipients of stem cell transplant have been conducted, the genetic factors that confer increased susceptibility to IA have yet to be validated. In light of the high fatality rate of IA, identification of such factors would improve prophylactic measures against not only IA but invasive infection by other mold species.

## References

[1] Kwon-Chung KJ, Bennett JE (1992) Medical mycology. Philadelphia: Lea & Febiger. 823 p.

[2] Latgé J-P (1999) *Aspergillus fumigatus* and aspergillosis. Clin Microbiol Rev 12: 310–350. 10194462 10.1128/cmr.12.2.310 PMC88920

[3] Latgé J-P (2001) The pathobiology of *Aspergillus fumigatus* . Trends Microbiol 9: 382–389. 11514221 10.1016/s0966-842x(01)02104-7

[4] Brakhage AA, Langfelder K (2002) Menacing mold: the molecular biology of *Aspergillus fumigatus* . Annu Rev Microbiol 56: 433–455. 12142473 10.1146/annurev.micro.56.012302.160625

[5] Dagenais TR, Keller NP (2009) Pathogenesis of *Aspergillus fumigatus* in invasive aspergillosis. Clin Microbiol Rev 22: 447–465. 19597008 10.1128/CMR.00055-08 PMC2708386

[6] Tekaia F, Latgé J-P (2005) *Aspergillus fumigatus*: saprophyte or pathogen? Curr Opin Microbiol 8: 385–392. 16019255 10.1016/j.mib.2005.06.017

[7] Hohl TM, Feldmesser M (2007) *Aspergillus fumigatus*: principles of pathogenesis and host defense. Eukaryot Cell 6: 1953–1963. 17890370 10.1128/EC.00274-07 PMC2168400

[8] Abad A, Fernandez-Molina JV, Bikandi J, Ramirez A, Margareto J, et al (2010) What makes *Aspergillus fumigatus* a successful pathogen? Genes and molecules involved in invasive aspergillosis. Rev Iberoam Micol 27: 155–182. 20974273 10.1016/j.riam.2010.10.003

[9] Kozakiewicz Z, Smith D (1994) Physiology of *Aspergillus* In: Smith JE, editor. Biotechnology handbooks - 7: *Aspergillus*. New York: Plenum Press. pp. 23–40.

[10] Jensen HL (1931) The fungus flora of the soil. Soil Science 31: 123–158.

[11] Gibbons JG, Beauvais A, Beau R, McGary KL, Latgé J-P, et al (2012) Global transcriptome changes underlying colony growth in the opportunistic human pathogen *Aspergillus fumigatus* . Eukaryot Cell 11: 68–78. 21724936 10.1128/EC.05102-11 PMC3255943

[12] Anastasi A, Varese GC, Marchisio VF (2005) Isolation and identification of fungal communities in compost and vermicompost. Mycologia 97: 33–44. 16389954 10.3852/mycologia.97.1.33

[13] Egel-Mitani M, Olson LW, Egel R (1982) Meiosis in *Aspergillus nidulans*: another example for lacking synaptonemal complexes in the absence of crossover interference. Hereditas 97: 179–187. 6761318 10.1111/j.1601-5223.1982.tb00761.x

[14] Sugui JA, Losada L, Wang W, Varga J, Ngamskulrungroj P, et al (2011) Identification and characterization of *Aspergillus fumigatus* ‘supermater’ pair. mBio 2: e00234–11 doi:10.1128/mBio.00234-11 22108383 10.1128/mBio.00234-11 PMC3225970

[15] Nierman WC, May G, Kim HS, Anderson MJ, Chen D, et al (2005) What the *Aspergillus* genomes have told us. Med Mycol 43 Suppl 1: S3–5. 16110785 10.1080/13693780400029049

[16] Frisvad JC, Samson RA (1990) Chemotaxonomy and morphology of *Aspergillus fumigatus* and related taxa. In: Samson RA, Pitt JI, editors. Modern concepts in *Penicillium* and *Aspergillus* classification. New York: Plenum Press. pp. 201–208.

[17] Sugui JA, Pardo J, Chang YC, Zarember KA, Nardone G, et al (2007) Gliotoxin is a virulence factor of *Aspergillus fumigatus*: *gliP* deletion attenuates virulence in mice immunosuppressed with hydrocortisone. Eukaryot Cell 6: 1562–1569. 17601876 10.1128/EC.00141-07 PMC2043361

[18] Burns C, Geraghty R, Neville C, Murphy A, Kavanagh K, et al (2005) Identification, cloning, and functional expression of three glutathione transferase genes from *Aspergillus fumigatus* . Fungal Genet Biol 42: 319–327. 15749051 10.1016/j.fgb.2005.01.001

[19] Tsai HF, Wheeler MH, Chang YC, Kwon-Chung KJ (1999) A developmentally regulated gene cluster involved in conidial pigment biosynthesis in *Aspergillus fumigatus* . J Bacteriol 181: 6469–6477. 10515939 10.1128/jb.181.20.6469-6477.1999 PMC103784

[20] Brakhage AA, Liebmann B (2005) *Aspergillus fumigatus* conidial pigment and cAMP signal transduction: significance for virulence. Med Mycol 43: S75–82. 16110796 10.1080/13693780400028967

[21] Taha MP, Pollard SJ, Sarkar U, Longhurst P (2005) Estimating fugitive bioaerosol releases from static compost windrows: feasibility of a portable wind tunnel approach. Waste Manag 25: 445–450. 15869988 10.1016/j.wasman.2005.02.013

[22] Beever RE, Dempsey GP (1978) Function of rodlets on the surface of fungal spores. Nature 272: 608–610. 148008 10.1038/272608a0

[23] Bayry J, Aimanianda V, Guijarro JI, Sunde M, Latgé J-P (2012) Hydrophobins—unique fungal proteins. PLoS Pathog 8: e1002700 doi:10.1371/journal.ppat.1002700 22693445 10.1371/journal.ppat.1002700 PMC3364958

[24] Ingham CJ, Kalisman O, Finkelshtein A, Ben-Jacob E (2011) Mutually facilitated dispersal between the nonmotile fungus *Aspergillus fumigatus* and the swarming bacterium *Paenibacillus vortex* . Proc Natl Acad Sci U S A 108: 19731–19736. 22106274 10.1073/pnas.1102097108 PMC3241745

[25] Cohen J, Postma DS, Douma WR, Vonk JM, De Boer AH, et al (2011) Particle size matters: diagnostics and treatment of small airways involvement in asthma. Eur Respir J 37: 532–540. 20595155 10.1183/09031936.00204109

[26] Reponen T, Willeke K, Ulevicius V, Reponen A, Grinshpun SA (1996) Effect of relative humidity on the aerodynamic diameter and respiratory deposition of fungal spores. Atmos Environ 30: 3967–3974.

[27] Wasylnka JA, Simmer MI, Moore MM (2001) Differences in sialic acid density in pathogenic and non-pathogenic *Aspergillus* species. Microbiology 147: 869–877. 11283283 10.1099/00221287-147-4-869

[28] Hospenthal DR, Kwon-Chung KJ, Bennett JE (1998) Concentrations of airborne *Aspergillus* compared to the incidence of invasive aspergillosis: lack of correlation. Med Mycol 36: 165–168. 9776829

[29] McDonagh A, Fedorova ND, Crabtree J, Yu Y, Kim S, et al (2008) Sub-telomere directed gene expression during initiation of invasive aspergillosis. PLoS Pathog 4: e1000154 doi:10.1371/journal.ppat.1000154 18787699 10.1371/journal.ppat.1000154 PMC2526178

[30] Sugui JA, Kim HS, Zarember KA, Chang YC, Gallin JI, et al (2008) Genes differentially expressed in conidia and hyphae of *Aspergillus fumigatus* upon exposure to human neutrophils. PLoS ONE 3: e2655 doi:10.1371/journal.pone.0002655 18648542 10.1371/journal.pone.0002655 PMC2481287

